# In Situ Electrochemical Atomic Force Microscopy Study of Interfacial Reactions on a Graphite Negative Electrode for Magnesium-Ion Batteries

**DOI:** 10.3390/ijms26146793

**Published:** 2025-07-15

**Authors:** Sungjae Yoon, Paul Maldonado Nogales, Sangyup Lee, Seunga Yang, Soon-Ki Jeong

**Affiliations:** 1 Department of Future Convergence Technology, Soonchunhyang University, Soonchunhyang-ro 22-gil, Sinchang-myeon, Asan-si 31538, Chungcheongnam-do, Republic of Korea; sjyoon@katech.re.kr (S.Y.); maldonado@sch.ac.kr (P.M.N.); 20237450@sch.ac.kr (S.L.); tmddk1107@sch.ac.kr (S.Y.); 2 Department of Energy Engineering, Soonchunhyang University, Soonchunhyang-ro 22-gil, Sinchang-myeon, Asan-si 31538, Chungcheongnam-do, Republic of Korea; 3 Advanced Energy Research Center, Soonchunhyang University, Soonchunhyang-ro 22-gil, Sinchang-myeon, Asan-si 31538, Chungcheongnam-do, Republic of Korea

**Keywords:** magnesium-ion battery, graphite electrode, solvated ion co-intercalation, solid electrolyte interphase formation, in situ atomic force microscopy

## Abstract

The cointercalation of solvated Mg^2+^ ions into graphite has typically been considered challenging because of concerns regarding the instability of the electrolyte and the potential for structural degradation. However, recent developments in electrolyte design suggest that this process may be reversible under appropriate conditions. In this study, the interfacial behavior of graphite in a magnesium-ion system was investigated using in situ electrochemical atomic force microscopy. Electrochemical tests in a triglyme-based electrolyte revealed a reversible capacity of 158 mAh g^−1^, attributed to the insertion of triglyme-solvated Mg^2+^ ions. Real-time surface imaging of highly oriented pyrolytic graphite revealed the formation of a passivating surface film during the initial cycle, along with nanoscale hill-like (~1 nm) and blister-like (~5 nm) structures, which were partially reversible and showed good correlation with the redox peaks observed in the cyclic voltammetry experiments, suggesting that the surface film enables Mg^2+^ transport while mitigating electrolyte decomposition. These findings demonstrate that stable co-intercalation of solvated Mg^2+^ ions is achievable in the early cycles in graphite and highlight the importance of interfacial engineering and solvation structures in the development of magnesium-ion batteries.

## 1. Introduction

Graphite, a layered allotrope of carbon, is widely used as a negative electrode material in lithium-ion batteries (LIBs) due to its ability to intercalate lithium ions between graphene layers reversibly via electrochemical redox reactions. This host–guest interaction relies on weak van der Waals forces, allowing ions to intercalate reversibly, which forms the basis of energy storage in many rechargeable battery systems. One of the key factors in ensuring cycling stability in LIBs is the formation of a solid electrolyte interphase (SEI), which passivates the graphite surface by preventing further electrolyte decomposition while allowing selective lithium-ion transport [1,2].

In ethylene carbonate (EC)-based electrolytes, SEI formation is often accompanied by partial solvent co-intercalation during the initial cycle, which contributes to the stabilization of the electrode–electrolyte interface [3,4]. In contrast, propylene carbonate (PC)-based electrolytes promote persistent co-intercalation of solvated lithium ions, which can result in exfoliation and structural degradation of the graphite matrix [5]. Therefore, EC-based electrolytes are preferred in commercial LIBs. Recent studies have demonstrated that under carefully controlled electrochemical conditions, such as optimized current densities or tailored electrolyte compositions, PC-solvated lithium ions can reversibly intercalate into graphite without inducing permanent structural damage [6,7].

These findings highlight the importance of understanding the solvated-ion intercalation behavior and its influence on interfacial stability, which is a critical problem in lithium-ion systems and emerging multivalent-ion batteries. Magnesium-ion batteries (MIBs) have attracted attention as promising next-generation energy storage systems because of the divalent nature of Mg^2+^ ions, which enables a higher charge carrier density per ion than monovalent ions such as Li^+^. Additionally, magnesium is naturally abundant and does not form dendrites during deposition, offering notable advantages in terms of cost and safety [8]. However, the use of metallic magnesium as a negative electrode is limited by the formation of ionically insulating passivation layers upon exposure to conventional electrolytes, which hinders reversible magnesium deposition and dissolution [9,10]. To address this limitation, alternative electrolyte systems such as Grignard reagents and organohaloaluminates have been developed, although they often exhibit poor anodic stability and cause corrosion of the cell components [11,12].

As an alternative to metallic magnesium, graphite has been explored as a negative electrode capable of reversibly intercalating solvated Mg^2+^ ions. Previous electrochemical studies employing Mg(TFSI)_2_ salts dissolved in polar solvents, such as dimethylformamide or mixed solvents composed of diglyme and dimethyl ether, have demonstrated the feasibility of inserting and extracting solvated magnesium ions from graphite layers under electrochemical control [13,14,15]. These results support the feasibility of graphite-based magnesium ion systems that do not rely on magnesium metal. However, the interfacial mechanisms governing SEI formation and surface morphology changes are poorly understood.

This knowledge gap hinders the rational design of stable magnesium-ion systems, where interfacial stability is critical for capacity retention and safety. To address this gap, we employed in situ electrochemical atomic force microscopy (ECAFM) to visualize interfacial changes on the basal plane of highly oriented pyrolytic graphite (HOPG) during the intercalation and deintercalation of solvated Mg^2+^ ions in a triglyme-based electrolyte. This technique enables the real-time observation of surface morphological changes during cyclic voltammetry (CV), providing insight into the reversibility of magnesium-ion insertion and the role of SEI-like interfacial films. The graphite electrode exhibited a capacity notably higher than those of previously reported electrodes and showed distinct surface features, including hill and blister structures. These findings enhance our understanding of the interfacial phenomena that govern solvated magnesium-ion intercalation and may guide the design of more stable and efficient MIB systems.

## 2. Results and Discussion

### 2.1. Electrochemical and Structural Evidence of Solvated Mg^2+^ Intercalation

**Figure 1:**
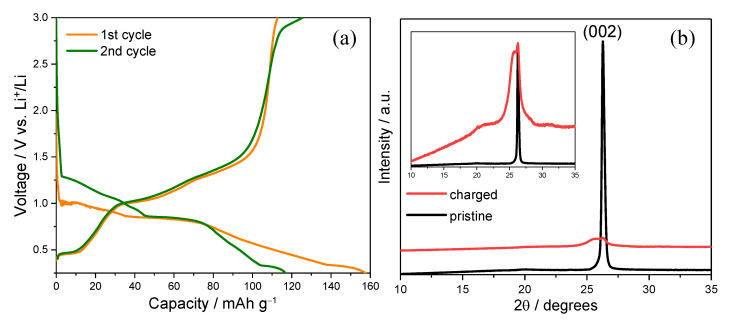
(**a**) Voltage profiles of the graphite composite electrode during the first and second cycles in 0.1 mol dm^−3^ Mg(ClO_4_)_2_/triglyme, showing an initial capacity loss of approximately 40 mAh g^−1^. (**b**) XRD patterns of the electrode before and after charging to 0.25 V. The (002) peak remains at ~26.5°, with noticeable broadening and reduced intensity. The inset shows the same XRD spectra normalized to the respective peak maxima to facilitate clearer comparison of peak shape and position.

Figure 1a shows the galvanostatic charge–discharge profiles of the graphite composite electrode during the first and second cycles in 0.1 mol dm^−3^ Mg(ClO_4_)_2_ dissolved in triglyme. In the first charge (intercalation), a sloping voltage profile is observed from the open-circuit voltage down to ~0.25 V, with no sharp plateaus. This behavior suggests solid-solution-type insertion of solvated Mg^2+^ ions. This was likely accompanied by increasing staging disorder within the graphite interlayers. The relatively broad voltage range and lack of distinct inflection points further support the interpretation that intercalation proceeds without forming well-ordered compounds [16]. Upon discharging (deintercalation), the capacity recovery is incomplete, resulting in an initial irreversible capacity loss of approximately 40 mAh g^−1^. This loss is likely attributable to parasitic reactions at the electrode–electrolyte interface, such as solvent decomposition and surface film formation, which are potentially analogous to the SEI observed in lithium-ion systems [17]. Such interfacial passivation may be critical in stabilizing the electrode surface and enabling subsequent reversible electrochemical reactions. This initial interfacial film formation is also responsible for the increased kinetic resistance and overpotential observed during the first intercalation cycle, which delays the voltage response and leads to a lower onset voltage compared to subsequent cycles. In the second cycle, the charge and discharge profiles become more symmetrical and reproducible, indicating improved reversibility of Mg^2+^ intercalation and extraction. The electrolyte used in this study consisted solely of 0.1 mol dm^−3^ Mg(ClO_4_)_2_ in triglyme, with no added lithium salt. Although lithium metal was employed as the counter electrode, the system contained no significant source of Li^+^ ions. Lithium pseudo-reference electrodes are reported to exhibit a stable positive potential offset of approximately +80 mV [18], meaning that the actual electrode potential during testing is estimated to be approximately 0.33–3.08 V vs. Li^+^/Li. Since this range lies entirely above the known Li^+^ intercalation window (0.0–0.25 V), and no Li^+^-specific voltage features were observed, the possibility of Li^+^ intercalation is considered negligible. The observed intercalation behavior is attributed to solvated Mg^2+^ ions. The current response and capacity retention suggest that the initial passivation layer effectively suppresses further electrolyte degradation while permitting selective ion transport [19]. These results confirm that the reversible intercalation of solvated Mg^2+^ ions can be achieved after establishing a stabilized interface in glyme-based electrolyte systems.

Following the electrochemical characterization, structural changes in the graphite electrode were investigated via XRD analysis, as shown in Figure 1b. The pristine electrode exhibits a sharp diffraction peak at 2θ ≈ 26.5°, corresponding to the (002) plane of well-ordered graphitic layers. After charging to 0.25 V in the first cycle, this peak shows significant broadening and a notable decrease in intensity, although its position remains largely unchanged. In typical ion intercalation systems, a shift in the (002) peak to lower angles indicates an expansion of the interlayer spacing due to the insertion of bare ions [20]. However, the absence of such a shift suggests that the Mg^2+^ ions were intercalated in a solvated form and likely still coordinated with the triglyme molecules. The peak broadening and intensity attenuation indicate partial structural disorder, which is consistent with the co-intercalation of bulky solvated species rather than bare Mg^2+^. These diffraction features support the hypothesis that the observed capacity in the electrochemical measurements originates from the reversible intercalation of solvated magnesium ions without collapse of the graphite framework. This behavior is similar to that observed in certain lithium systems under cointercalation conditions [21]. It highlights the unique role of solvation in stabilizing the interlayer insertion processes in multivalent-ion battery systems.

These insights motivated us to examine the dynamic interfacial evolution in real time using in situ ECAFM coupled with CV, as detailed in Section 2.2.

### 2.2. CV Analysis of HOPG Before In Situ Surface Imaging

**Figure 2:**
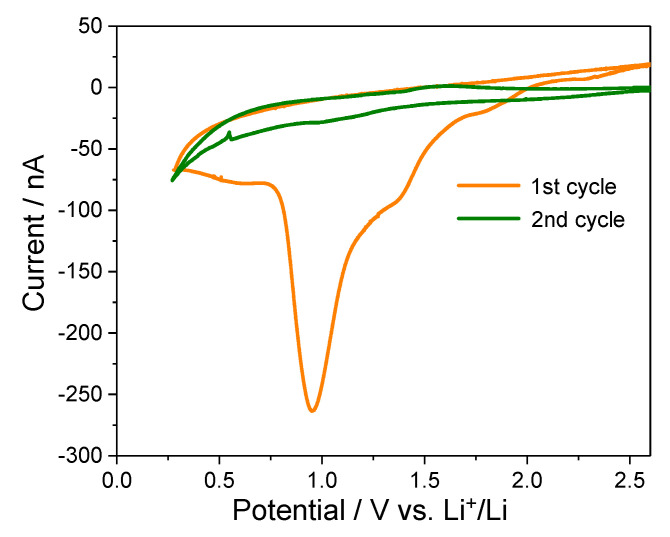
Cyclic voltammograms of the HOPG electrode during in situ ECAFM in 0.1 mol dm^−3^ Mg(ClO_4_)_2_/triglyme. A reduction peak near 1.0 V appears only in the first cycle, whereas a second peak at ~0.25 V persists in the subsequent scans.

To complement the bulk-level electrochemical and structural analyses, in situ ECAFM was employed to visualize directly the interfacial changes associated with solvated Mg^2+^ intercalation. This technique simultaneously captures the electrochemical responses and nanoscale morphological changes. HOPG was selected as the model electrode to ensure reliable imaging owing to its atomically flat basal plane [22]. Figure 2 presents the CV profiles of the HOPG electrode obtained during the first two cycles in 0.1 mol dm^−3^ Mg(ClO_4_)_2_/triglyme electrolyte. During the first reduction sweep, a pronounced cathodic current peak is observed near 1.0 V (vs. Li^+^/Li), which disappears in the subsequent cycle. This irreversible feature suggests a one-time interfacial reaction, most likely associated with electrolyte decomposition and surface film formation. This behavior is consistent with the SEI formation behavior widely reported in lithium-ion systems [23] and suggests that a similar passivation mechanism may operate in magnesium-ion systems, despite the differences in the solvation and charge properties of Mg^2+^.

In contrast, a distinct and reproducible cathodic response appears near 0.25 V in the first and second cycles. This feature is attributed to the co-intercalation of triglyme-solvated Mg^2+^ ions into the graphite interlayers, which is consistent with the reversible redox behavior observed in the galvanostatic charge–discharge experiments. The persistence of this response implies that, although electron tunneling may be suppressed by the passivation film, Mg^2+^ ions can still migrate through the film and intercalate into the graphite galleries. This aligns with the widely accepted dual functions of SEI-like interphases: electron blocking and ion conduction [15].

The electrochemical characteristics identified in the CV curves correlate well with the charge–discharge profiles and XRD data presented in Figure 2. Specifically, the irreversible peak near 1.0 V aligns with the initial capacity loss and the onset of structural disorder, and the sustained redox activity near 0.25 V corroborates the hypothesis of reversible solvated-ion intercalation. These observations support the view that the passivating interfacial film formed during the first cycle is crucial in enabling reversible Mg^2+^ insertion reactions. The detailed correlation between these voltammetric responses and accompanying nanoscale surface morphological changes is discussed in Section 2.3.

### 2.3. Surface Evolution on HOPG Basal Plane During CV Cycling

**Figure 3:**
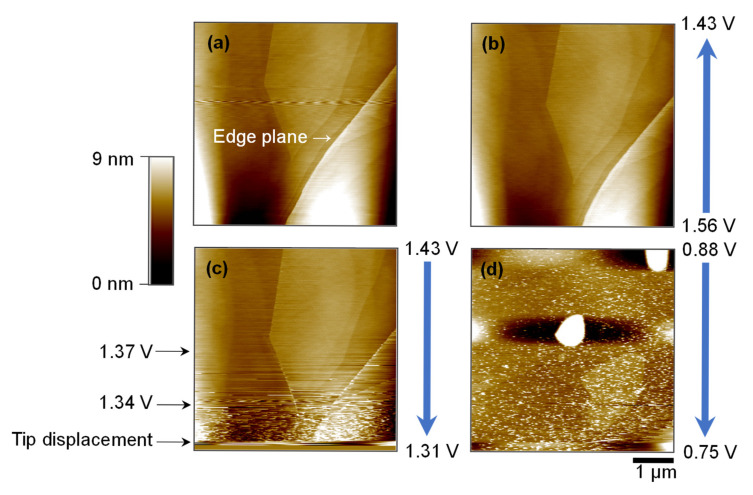
AFM images (5 × 5 µm^2^) of the HOPG basal plane surface during the first CV cycle in 0.1 mol dm^−3^ Mg(ClO_4_)_2_/triglyme. (**a**) Pristine surface at 2.9 V. (**b**) No significant changes are observed between 1.56 and 1.43 V. (**c**) Surface roughening and granular features appear between 1.43 and 1.31 V. (**d**) More pronounced modification is observed between 0.88 and 0.75 V. Note: Horizontal line features observed in the image are characteristic of in situ ECAFM under liquid electrolyte conditions and may arise from tip–surface interactions influenced by electrolyte decomposition, film formation, or transient interfacial reactions. These features are commonly reported in the ECAFM literature and have been intentionally retained to preserve the integrity of the surface information.

Figure 3 illustrates the morphological evolution of the HOPG basal plane as a function of applied electrode potential during CV cycling in 0.1 mol dm^−3^ Mg(ClO_4_)_2_/triglyme electrolyte. Owing to its atomically smooth surface, HOPG enables the nanoscale visualization of interfacial processes that occur during the intercalation of solvated Mg^2+^ ions [24]. Before electrochemical cycling, the basal surface (Figure 3a) exhibits well-defined step edges and atomically flat terraces, characteristic of pristine graphite. These features are consistent with those of an unmodified surface and serve as a baseline for detecting potentially induced transformations.

As the applied potential is decreased from the open-circuit voltage to ~1.56 V and subsequently 1.43 V (Figure 3b), the surface remains largely unchanged, indicating minimal electrochemical activity at these relatively high potentials. However, upon further reduction to 1.31–1.34 V (Figure 3c), significant surface roughening begins to appear. The fine granular features and increased noise in the lower portion of the image suggest the formation of a surface film, likely resulting from electrolyte decomposition [25]. This transition correlates closely with the cathodic peak near 1.0 V observed in the CV profile (Figure 2), supporting the hypothesis that film formation and interfacial passivation begin within this potential window.

At a more negative potential of 0.75 V (Figure 3d), the surface exhibits more pronounced topographical changes. A dense distribution of hill-like or blister structures appears, indicating substantial morphological alteration. Although the lateral position of the AFM tip may have shifted slightly during scanning, the observed features reflect genuine surface modifications rather than imaging artifacts. The increased roughness and particle-like accumulation indicate continued film growth and possible co-intercalation effects involving solvated Mg^2+^ ions [26]. Notably, these changes occur even on the typically inert basal plane, highlighting the pronounced surface reactivity induced under reducing conditions [27].

These results demonstrate that interfacial reactions—initiated at approximately 1.3 V and intensified at lower potentials—lead to electrochemical and morphological transformations of the graphite surface. The evolution of the surface structure, observed in parallel with the CV features, provides direct evidence for the formation of SEI-like films under potential-dependent conditions and underscores the dynamic nature of the electrode–electrolyte interface in glyme-based magnesium-ion systems. Although the above observations reflect early-stage passivation dynamics, they do not fully capture the interfacial morphology during reversible Mg^2+^ co-intercalation. Section 2.4 presents detailed AFM imaging results that reveal how solvated Mg^2+^ ions modulate the surface structure over the entire CV cycle.

### 2.4. Morphological Evidence of Reversible Co-Intercalation: Hill and Blister Formation

**Figure 4:**
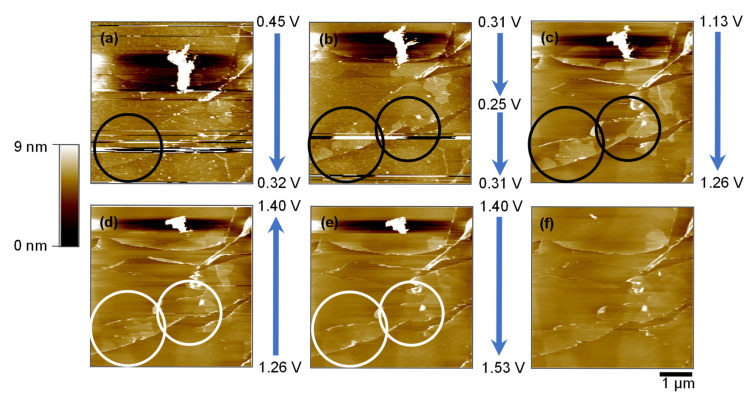
AFM images (5 × 5 µm^2^) of the HOPG basal plane surface during the first CV cycle in 0.1 mol dm^−3^ Mg(ClO_4_)_2_/triglyme. Images were acquired at (**a**) 0.45–0.32 V, (**b**) 0.25–0.31 V, (**c**) 1.13–1.26 V, (**d**) 1.26–1.40 V, and (**e**) 1.40–1.53 V. Hill-like features form near the edge plane during the reduction sweep (**b**) and gradually diminish during the reverse sweep (**c**–**e**). (**f**) Final surface at 2.9 V after the first cycle shows no major irreversible deformation. Note: A bright spot appears intermittently in the top region of images (**a**–**e**), which disappears in (**f**). This feature is attributed to a transient imaging artifact (e.g., tip contamination) and is not associated with a persistent morphological change on the sample surface. Horizontal line features are retained as described in Figure 3. Black and white circles indicate the localized regions where hill-like structures appear and then diminish across the potential sweep.

Figure 4 presents a sequential series of AFM images that illustrate the morphological evolution of the HOPG basal plane during CV in the potential range of 0.25–1.53 V (vs. Li^+^/Li). These images provide direct nanoscale evidence of the interfacial changes associated with solvated Mg^2+^ intercalation and deintercalation.

At the initial potential of 0.45 V (Figure 4a), the HOPG basal surface remains smooth, showing well-defined atomic terraces and step edges. As the potential decreases to 0.25 V and then returns to 0.31 V (Figure 4b), distinct hill-like protrusions emerge in proximity to the edge plane (black circles), which are absent in the initial state. The localization of these features near the edge sites, which are known to be more electrochemically active than the basal regions, suggests that solvated Mg^2+^ ions intercalate preferentially via the edge plane [28]. Such surface modifications are analogous to the hill structures reported in LIB systems during the cointercalation of solvated Li^+^ species, wherein localized volumetric expansion occurs owing to the insertion of bulky solvated ions [29].

As the potential is gradually reversed from 1.26 V to 1.53 V (Figure 4c–e), the hill structures diminish in prominence. This gradual disappearance indicates the partial deintercalation of the triglyme–Mg^2+^ complexes. The final image at 1.53 V (Figure 4f) reveals a surface morphology closely resembling the pre-cycling state, implying that deformation is largely reversible under the experimental conditions. While tip-induced deformation is always a consideration in contact-mode AFM, the hill- and blister-like features observed in this study appeared selectively within specific potential ranges and exhibited partial reversibility upon voltage reversal. No such features were detected at open-circuit or in the absence of applied potential, supporting the interpretation that these structures result from electrochemical processes, rather than mechanical interaction between the AFM tip and the surface. The morphological reversibility complements the electrochemical data in Figure 2, where reversible current responses were observed at ~0.25 V in both cycles. AFM images were intentionally presented without flattening or post-processing to preserve the original interfacial morphology. Line features reflect intrinsic scan variation under in situ conditions and were intentionally retained to avoid misrepresenting electrochemically relevant structures.

**Figure 5:**
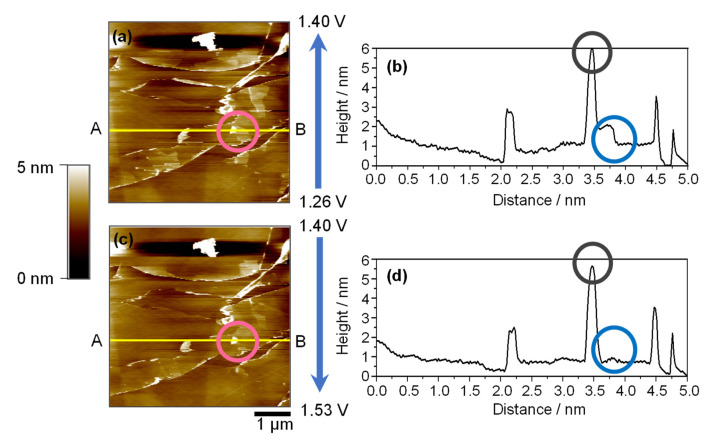
AFM images (10 × 10 µm^2^) and corresponding height profiles of the HOPG basal plane surface during the first CV cycle in 0.1 mol dm^−3^ Mg(ClO_4_)_2_/triglyme. Topographic images were acquired at (**a**) 1.26–1.40 V and (**c**) 1.40–1.53 V. Line profiles (**b**,**d**) correspond to the yellow lines in (**a**,**c**), respectively. The pink circle highlights a representative hill-like structure that disappears during anodic cycling. Blue and black circles in the height profiles emphasize persistent blister-like protrusions (~5 nm) that remain unchanged throughout the potential sweep. Note: Horizontal line features are retained as described in Figure 3.

Figure 5 provides a complementary quantitative analysis of these surface changes, including topographic maps and height profiles before (Figure 5a,b) and after (Figure 5c,d) surface recovery. The pink circle highlights a representative hill structure that disappears during anodic cycling. Height profile analysis along the same cross-section (yellow lines A–B) revealed an elevation of ~1 nm before disappearance, confirming the nanometric scale of these transient features. This height is consistent with previously reported hill-like structures in EC-based lithium systems [30].

In addition to the hill structures, persistent blister-like protrusions are observed as bright dots in both images (Figure 5a,c), which are further emphasized in the profiles by the blue circles. These features, with heights of approximately 5 nm, do not change significantly throughout the potential sweep, suggesting the robustness of their structure. Compared to analogous blisters in carbonate-based lithium-ion systems, which often range from 15 to 500 nm, these features are significantly smaller, likely owing to differences in the solvation environments and ionic radii [31]. Using an ether-based triglyme as a solvent may reduce the steric strain and volume expansion during co-intercalation, contributing to a more benign form of surface distortion.

Collectively, these findings demonstrate that the triglyme–Mg^2+^ system supports a relatively mild yet reversible intercalation process, leading to nanoscale surface modifications, specifically hill and blister formation, without catastrophic exfoliation or irreversible damage to the graphite host. The observed phenomena highlight the critical influence of the solvation chemistry and surface structure in governing the interfacial behavior of multivalent-ion battery systems.

### 2.5. Comparison of Solvated Ion Insertion Effects with LIB Systems

To contextualize the interfacial behavior of the triglyme-solvated Mg^2+^ ions observed in this study, comparisons were made with previously reported results for lithium-ion systems employing carbonate-based electrolytes. In LIBs using EC, co-intercalation of solvated Li^+^ ions into graphite typically leads to hill-like surface protrusions on the basal plane, with characteristic heights ranging from 0.8 to 1.0 nm [32]. The present results reveal that analogous hill structures are also formed during Mg^2+^ intercalation in triglyme, suggesting a similar co-intercalation mechanism via the edge plane of graphite.

However, notable distinctions were evident in the blister formation behavior. In the magnesium system, the blister-like protrusions that persisted throughout the voltage sweep were approximately 5 nm in height. This is substantially smaller than the blister dimensions reported for EC-based LIB systems, which often range from 15 to more than 100 nm [33]. These differences likely reflect two key factors: (i) the divalent nature of Mg^2+^, which may enhance Coulombic stabilization and limit volumetric distortion, and (ii) the linear molecular geometry of the ether-based triglyme, which imposes a reduced steric strain compared to cyclic carbonates such as EC. These solvation and ionic effects jointly modulate the interfacial stress and structural deformation during co-intercalation.

Further insights can be obtained by considering the interlayer expansion trends. Previous studies have reported graphite interlayer spacings of approximately 1.116 and 1.165 nm for diglyme-solvated Li^+^ and Na^+^, respectively [34]. In the present study, the co-intercalation of Mg^2+^ solvated in triglyme led to an estimated interlayer distance of ~1.2 nm, representing a ~60% increase over the pristine value of 0.335 nm. This finding supports the hypothesis that the triglyme solvation shell remains intact during intercalation and is accommodated within the expanded graphite galleries.

**Figure 6:**
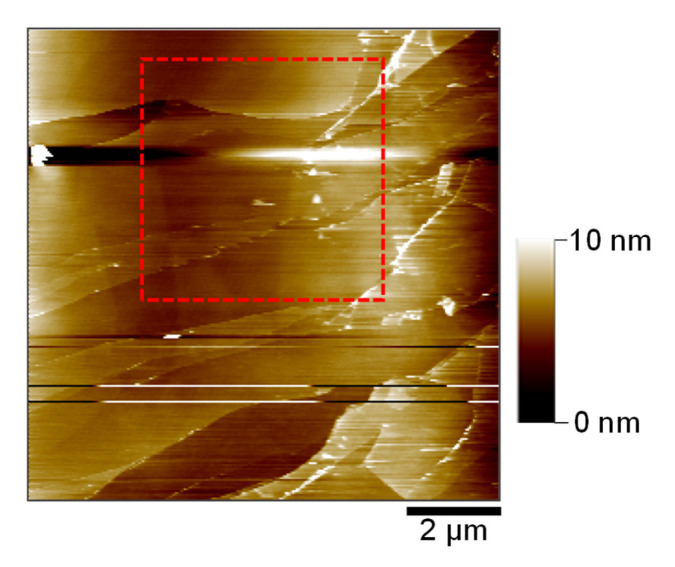
AFM image (10 × 10 µm^2^) of the HOPG basal plane surface obtained at 2.9 V after the first CV cycle in 0.1 mol dm^−3^ Mg(ClO_4_)_2_/triglyme. The red dashed box indicates the same region observed during in situ imaging in Figure 4. Preserved step edges and moderate roughness suggest limited irreversible surface changes following solvated Mg^2+^ intercalation and de-intercalation. Note: Horizontal line features are retained as described in Figure 3.

Figure 6 shows the AFM topography of the HOPG basal surface after a complete CV cycle. The red dashed square indicates the area selected for the detailed surface evolution analysis. Despite repeated electrochemical cycling, HOPG retains its well-defined step edges and exhibits only moderate roughening. The absence of severe blistering or irreversible deformation, commonly observed in carbonate-based LIB systems [35], suggests that solvated Mg^2+^ intercalation into the triglyme induces only mild and reversible morphological changes. This comparison highlights the unique interfacial behavior of the Mg^2+^/triglyme system. It suggests that the rational selection of the solvation environment may mitigate structural degradation commonly seen in carbonate-based systems.

Overall, this comparative analysis underscores the significance of solvation design in achieving stable interfacial behavior in multivalent systems. These findings emphasize the critical role of ion–solvent interactions in dictating interfacial phenomena during intercalation. The relatively stable surface morphology and structural reversibility demonstrated by the Mg^2+^/triglyme system underscore the potential of ether-based electrolytes for developing durable and efficient multivalent-ion batteries. Future efforts focusing on tuning the solvation structure and host–guest chemistry may further advance the design of cointercalation-capable systems for next-generation energy storage applications.

## 3. Materials and Methods

### 3.1. Electrode Fabrication and Electrochemical Testing

Natural graphite powder (CGB-10, Nippon Graphite Industries, Ltd., Tokyo, Japan) was used as the active material, and poly(vinylidene fluoride) (PVdF, Mw ≈ 534,000; Sigma-Aldrich, St. Louis, MO, USA) served as the binder. The two components were mixed in a 9:1 weight ratio to form a uniform slurry, which was cast onto the copper foil (18 μm, UACJ Corporation, Tokyo, Japan) using a doctor blade. The coated electrodes were dried in a vacuum oven (OV-11, Jeio Tech, Daejeon, Republic of Korea) at 80 °C for 12 h. The electrolyte was prepared by dissolving magnesium perchlorate (Mg(ClO_4_)_2_, ≥99%, Sigma-Aldrich, St. Louis, MO, USA) in battery-grade triethylene glycol dimethyl ether (triglyme, ENCHEM, Cheonan, Republic of Korea) to a concentration of 0.1 mol dm^−3^. All electrolyte solutions were stored in an argon-filled glove box (SK-G1200, Three-Shine Inc., Daejeon, Republic of Korea) maintained at a dew point below −70 °C to prevent moisture contamination.

The electrochemical measurements were performed using CR2032-type coin cells (MTI Corporation, Richmond, CA, USA) assembled in an argon-filled glovebox. The graphite composite electrode served as the working electrode, and lithium metal foil (500 μm, Honjo Metal Co., Ltd., Osaka, Japan) was used as the counter and reference electrodes. The potential of the lithium reference electrode is stabilized by the spontaneous formation of an SEI, which effectively inhibits electron transfer to Mg^2+^ ions and suppresses their reduction. The reliability of Li pseudo-reference electrodes in magnesium-ion systems has been previously validated in electrochemical studies using ferrocene internal standards [18]. Charge–discharge tests were carried out using a battery cycler (WBCS 3000, WonATech, Seoul, Republic of Korea), applying a current corresponding to a C-rate of 0.01 based on a theoretical capacity of 744 mAh g^−1^. The cells were cycled between 0.25 and 3 V (vs. Li^+^/Li), after a 10 h rest period following cell assembly. Structural changes in the graphite electrodes before and after cycling were evaluated using X-ray diffraction (XRD) with a benchtop diffractometer (MiniFlex 600, Rigaku Corp., Tokyo, Japan) equipped with a Cu Kα source (λ = 0.15406 nm, 40 kV, 15 mA). The XRD measurements were conducted at the Advanced Energy and Display Materials Analysis Center of Soonchunhyang University using equipment registered under the identifier NFEC-2018-12-247471.

### 3.2. ECAFM Measurements

**Figure 7:**
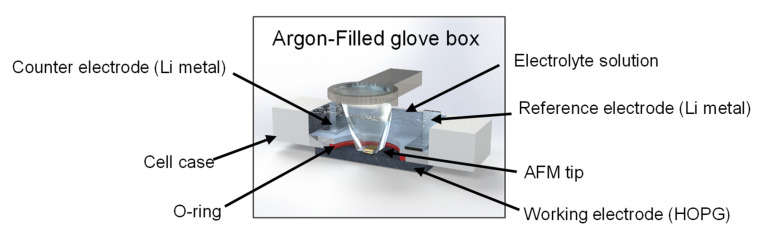
Schematic of the in situ three-electrode ECAFM cell, consisting of an HOPG working electrode, lithium metal counter/reference electrodes, and an electrolyte sealed with a Viton O-ring. Measurements were conducted inside an argon-filled glove box (dew point −70 °C).

Freshly cleaved HOPG (ZYH grade, mosaic spread: 3.5° ± 1.5°, Probes Korea, Daejeon, Republic of Korea) was used as a model electrode for CV and ECAFM measurements. The exposed surface area was confined to 1.2 cm^2^ using a Viton O-ring (DuPont, Wilmington, DE, USA) to ensure that only the basal plane was in contact with the electrolyte solution. The electrolyte was prepared by dissolving magnesium perchlorate (Mg(ClO_4_)_2_, ≥99%, Sigma-Aldrich, St. Louis, MO, USA) in battery-grade triethylene glycol dimethyl ether (triglyme, ENCHEM, Suwon, Republic of Korea) to a concentration of 0.1 mol dm^−3^. The water content of the electrolyte was maintained below 20 ppm. Lithium metal foil (500 μm thick, Honjo Metal Co., Ltd., Osaka, Japan) was used as the counter and reference electrodes, and all potentials were measured vs. Li^+^/Li. A custom-built electrochemical AFM cell was used for these measurements; its configuration is shown in Figure 7.

In situ ECAFM was performed using a PicoScan 2100 AFM system (Molecular Imaging, Tempe, AZ, USA) operated in the contact mode with a silicon nitride cantilever (OMCL-TR800PSA-1, Olympus Corp., Tokyo, Japan). This setup enabled the simultaneous acquisition of CV data and high-resolution surface images during electrochemical cycling. CV scans were conducted from the open-circuit potential down to 0.25 V (vs. Li^+^/Li) at a sweep rate of 0.5 mV s^−1^. All in situ ECAFM measurements were performed in an argon-filled glove box (SK-G1200, Three-Shine Inc., Daejeon, Republic of Korea) maintained at a dew point of −70 °C to prevent moisture contamination.

## 4. Conclusions

By integrating electrochemical, structural, and nanoscale imaging techniques, we elucidated the underlying interfacial dynamics governing solvated Mg^2+^ intercalation in graphite electrodes. During the first reduction cycle in CV, distinct interfacial changes were observed on the HOPG basal surface, such as surface film formation, hill-like protrusions, and blister structures. A pronounced reduction current near 1.0 V, that vanished in subsequent cycles, indicated the formation of a passivating interfacial layer, likely analogous to an SEI, which effectively suppressed further electrolyte decomposition. XRD and AFM analyses confirmed that triglyme-solvated Mg^2+^ ions were co-intercalated into graphite, resulting in reversible interlayer expansion without inducing structural collapse of the graphite matrix. Morphological analysis revealed that hill-like features (~1 nm in height) were observed near the edge plane at low potentials and largely disappeared upon reversal of the sweep, indicating a reversible cointercalation/deintercalation process. In contrast, blister-like protrusions (~5 nm high) persisted throughout the cycle, reflecting localized surface stress, but limited structural disruption compared to lithium systems using carbonate solvents. These findings demonstrate that the surface film formed during initial cycling played a dual role, simultaneously preventing further electrolyte decomposition and enabling selective Mg^2+^ transport. The observed interfacial stability and reversibility underscore the importance of the solvation structure and electrolyte formulation in enabling efficient Mg-ion intercalation in the initial stages of cycling. To fully understand the long-term morphological stability and degradation pathways of graphite electrodes in Mg-ion systems, future studies should include ex situ or operando surface imaging after extended cycling (e.g., 100 or more cycles), which would complement the early-stage insights provided by in situ ECAFM. Overall, this study offers mechanistic insights into the insertion of solvated ions into multivalent systems and highlights the key design considerations for future MIB technologies. While this study focused on real-time morphological analysis, future investigations combining in situ Fourier-transform infrared spectroscopy or air-sensitive techniques such as X-ray photoelectron spectroscopy or energy-dispersive X-ray spectroscopy would be valuable for identifying the chemical composition of the passivating interphase and for providing direct evidence of solvated Mg^2+^ intercalation. Overall, this study offers mechanistic insights into the insertion of solvated ions into multivalent systems and highlights the key design considerations for future MIB technologies.

## Data Availability

Data is contained within the article.

## References

[1] Zuo X., Zhu J., Müller-Buschbaum P., Cheng Y. (2017). Silicon based lithium-ion battery anodes: A chronicle perspective review. Nano Energy.

[2] Choi J., Aurbach D. (2016). Promise and reality of post-lithium-ion batteries with high energy densities. Nat. Rev. Mater..

[3] Li Y., Lu Y., Adelhelm P., Titirici M.M., Hu Y.S. (2019). Intercalation chemistry of graphite: Recent advances and future prospects. Chem. Soc. Rev..

[4] Jache B., Binder J.O., Abe T., Adelhelm P. (2016). Layered FeS_2_ as intercalation cathode for rechargeable sodium batteries. Phys. Chem. Chem. Phys..

[5] Wang G., Yu M., Feng X. (2021). Two-dimensional materials and their derived architectures for advanced sodium batteries. Chem. Soc. Rev..

[6] Song H.-Y., Jeong S.-K. (2018). Electrochemical Solvent Cointercalation into Graphite in Propylene Carbonate-Based Electrolytes: A Chronopotentiometric Characterization. J. Anal. Methods Chem..

[7] Kim H., Lim K., Yoon G., Park J., Lim H.D., Sung Y.E., Kang K. (2017). Time-resolved observation of lithium intercalation into graphite using in-situ EC-AFM. Adv. Energy Mater..

[8] Prabakar S., Ikhe A., Park W., Chung K.Y., Park H., Ahn D., Kwak J., Sohn K.S., Pyo M. (2019). Magnesium-based battery systems: Current state and challenges. Adv. Sci..

[9] Ling C., Banerjee D., Matsui M. (2012). Study of the electrochemical deposition behavior of Mg in non-aqueous electrolytes. Electrochim. Acta.

[10] Yoo H.D., Shterenberg I., Gofer Y., Gershinsky G., Pour N., Aurbach D. (2013). Mg rechargeable batteries: An on-going challenge. Energy Environ. Sci..

[11] Gregory T., Hoffman R., Winterton R. (1990). Nonaqueous electrochemistry of magnesium. J. Electrochem. Soc..

[12] Aurbach D., Lu Z., Schechter A., Gofer Y., Gizbar H., Turgeman R., Cohen Y., Moshkovich M., Levi E. (2000). Prototype systems for rechargeable magnesium batteries. Nature.

[13] God C., Bitschnau B., Kapper K., Lenardt C., Schmuck M., Mautner F.A., Koller S. (2017). Glyme-based electrolytes enabling magnesium intercalation into graphite. RSC Adv..

[14] Kim D., Jung S., Ha S., Kim Y., Park Y., Ryu J., Han Y., Lee K. (2018). Solvated magnesium ion intercalation into graphite from diglyme-based electrolyte. Chem. Mater..

[15] Winter M., Besenhard J.O., Spahr M.E., Novák P. (1998). Insertion electrode materials for rechargeable lithium batteries. Adv. Mater..

[16] Shimizu M., Nakahigashi A., Arai S. (2021). Intercalation/deintercalation of solvated Mg^2+^ into/from graphite interlayers. Phys. Chem. Chem. Phys..

[17] Ponrouch A., Verrelli R., Barda D., Frontera C., Palacín M.R. (2018). Electrochemical intercalation of calcium and magnesium in TiS_2_: Fundamental studies related to multivalent battery applications. Chem. Mater..

[18] Tchitchekova D.S., Monti D., Johansson P., Barde F., Randon-Vitanova A., Palacín M.R., Ponrouch A. (2017). On the Reliability of Half-Cell Tests for Monovalent (Li^+^, Na^+^) and Divalent (Mg^2+^, Ca^2+^) Cation Based Batteries. J. Electrochem. Soc..

[19] Tan S., Xiong F., Wang J., An Q., Mai L. (2020). Crystal regulation towards rechargeable magnesium battery cathode materials. Mater. Horiz..

[20] Mizuno Y., Okubo M., Hosono E., Kudo T., Honma I. (2013). Electrochemical Mg^2+^ intercalation into a bimetallic CuFe Prussian blue analog in aqueous electrolytes. J. Mater. Chem. A.

[21] Lee H.J., Shin J., Choi J.W. (2018). Intercalated water and organic molecules for electrode materials of rechargeable batteries. Adv. Mater..

[22] Jeong S.K., Inaba M., Mogi R., Iriyama Y., Abe T., Ogumi Z. (2001). Surface film formation on a graphite negative electrode in lithium-ion batteries: Atomic force microscopy study on the effects of film-forming additives in propylene carbonate solutions. Langmuir.

[23] Zhu H., Russell J.A., Fang Z., Barnes P., Li L., Efaw C.M., Daniel C. (2021). In situ analysis of solid electrolyte interphase formation and evolution on highly oriented pyrolytic and disordered graphite negative electrodes in lithium-ion batteries. Small.

[24] Chen Y., Wu W., Gonzalez-Munoz S., Forcieri L., Yang W., Reece M.J., Brett D.J.L., Shearing P.R. (2023). Nanoarchitecture factors of solid electrolyte interphase formation via 3D nano-rheology microscopy and surface force-distance spectroscopy. Nat. Commun..

[25] Jeong S.K., Inaba M., Iriyama Y., Abe T., Ogumi Z. (2003). AFM study of surface film formation on a composite graphite electrode in lithium-ion batteries. J. Power Sources.

[26] Mahmood A., Bai Z., Wang S., Lei Y., Wang S. (2025). Enabling high-performance multivalent metal-ion batteries: Current advances and future prospects. Chem. Soc. Rev..

[27] Hoane A.G., Zheng Q., Maldonado N.D., Lutz D.M., Shao-Horn Y. (2024). Impact of multivalent cations on interfacial layering in water-in-salt electrolytes. ACS Appl. Energy Mater..

[28] Domi Y., Ochida M., Tsubouchi S., Yamada K., Momma T. (2012). Electrochemical AFM observation of the HOPG edge plane in ethylene carbonate-based electrolytes containing film-forming additives. J. Electrochem. Soc..

[29] Fukutsuka T., Kokumai R., Song H.Y., Abe T. (2016). In-situ AFM observation of surface morphology of highly oriented pyrolytic graphite in propylene carbonate-based electrolyte solutions containing lithium and bivalent cations. J. Electrochem. Soc..

[30] Inaba M., Kawatate Y., Funabiki A., Jeong S.K., Abe T., Ogumi Z. (1999). STM study on graphite/electrolyte interface in lithium-ion batteries: Solid electrolyte interface formation in trifluoropropylene carbonate solution. Electrochim. Acta.

[31] Danis L., Gateman S.M., Kuss C. (2017). Nanoscale measurements of lithium-ion battery materials using scanning probe techniques. ChemElectroChem.

[32] Zhang Z., Said S., Smith K., Jervis R., Shearing P. (2021). Characterizing batteries by in situ electrochemical atomic force microscopy: A critical review. Adv. Energy Mater..

[33] Inaba M., Siroma Z., Kawatate Y., Funabiki A., Ogumi Z. (1997). Electrochemical STM study on lithium intercalation and surface morphology changes of graphite electrode in EC-based electrolytes. J. Power Sources.

[34] Dini D., Cognigni F., Passeri D., Rossi M., Rinaldi D. (2021). Multiscale characterization of Li-ion batteries through the combined use of atomic force microscopy and X-ray microscopy and considerations for a correlative analysis approach. J. Electrochem. Soc..

[35] Inaba M., Jeong S.K., Ogumi Z. (2011). In situ scanning probe microscopy of interfacial phenomena in batteries. Interface.

